# Eye-movement replay supports episodic remembering

**DOI:** 10.1098/rspb.2022.0964

**Published:** 2022-06-29

**Authors:** Roger Johansson, Marcus Nyström, Richard Dewhurst, Mikael Johansson

**Affiliations:** ^1^ Department of Psychology, Lund University, Lund, Sweden; ^2^ Humanities Lab, Lund University, Lund, Sweden; ^3^ Interacting Minds Centre, Århus University, Aarhus, Denmark

**Keywords:** eye movements, episodic memory, replay, scanpaths, reinstatement

## Abstract

When we bring to mind something we have seen before, our eyes spontaneously unfold in a sequential pattern strikingly similar to that made during the original encounter, even in the absence of supporting visual input. Oculomotor movements of the eye may then serve the opposite purpose of acquiring new visual information; they may serve as self-generated cues, pointing to stored memories. Over 50 years ago Donald Hebb, the forefather of cognitive neuroscience, posited that such a sequential *replay* of eye movements supports our ability to mentally recreate visuospatial relations during episodic remembering. However, direct evidence for this influential claim is lacking. Here we isolate the sequential properties of spontaneous eye movements during encoding and retrieval in a pure recall memory task and capture their encoding-retrieval overlap. Critically, we show that the fidelity with which a series of consecutive eye movements from initial encoding is sequentially retained during subsequent retrieval predicts the quality of the recalled memory. Our findings provide direct evidence that such *scanpaths* are replayed to assemble and reconstruct spatio-temporal relations as we remember and further suggest that distinct scanpath properties differentially contribute depending on the nature of the goal-relevant memory.

## 1. Background

Episodic memory refers to our ability to mentally travel back in time to relive past experiences in vivid detail [1]. The formation of coherent episodic memories critically hinges upon the binding of spatio-temporal relationships into a context, which predominantly depends on how we visually ‘sample’ the world when we act upon it via eye movements [2,3]. Eye movements unfold in sequences of fixations and saccades, where fixations are the brief moments that allow us to sample visual information, and saccades are the rapid movements that occur from one fixation point to another. Although only a limited amount of information can be processed at each fixation point, a sequence of consecutive fixations and saccades can effectively bind multiple inter-related episodic details together, allowing us to encode a memory representation of the event as a whole [4–12]. Thus, our visual sampling of the world is highly predictive of the content and quality of episodic memory formation [6–12]. Spontaneous eye movements also occur during episodic recollections, i.e. when the previously encoded event information is internalized mentally in the *absence of supporting visual input*. These have been demonstrated to broadly reproduce the gaze patterns that were established during encoding [13–19]. A prominent view holds that such gaze reinstatements actively support episodic remembering [20–22] and that the unfolding sequence of connected eye movements may serve to reconstruct episodic memories across time and space [13,14,22–26]. This claim receives support from previous research showing that the extent of spatial encoding-retrieval overlaps in gaze patterns positively correlates with retrieval performance [14,27,28], and with neural reactivation during retrieval [29,30]. Extending such findings, it has been demonstrated that the likelihood of successful remembering increases when gaze locations during recall are directly manipulated to overlap with those from encoding [31–35]. While these findings clearly show that where you look has important consequences for what you remember, they are all related to *static gaze patterns in space* and do not take into account how *dynamic sequences* of connected eye-movements progress over space and time. Also, active manipulations of gaze direction eliminate the spatio-temporal dynamics that are essential properties of free viewing [36]. Although research on *recognition* memory has shown that a temporal reinstatement of gaze patterns can be beneficial when discriminating between novel and previously encountered stimuli [37,38] (but see [39]), there is to date no direct evidence for the influential claim that the sequential *replay* of eye movements serves to facilitate pure episodic reconstruction in the absence of visual input. To address this fundamental question, it is necessary to go beyond static gaze patterns in space and examine how the sequential replay of connected eye movements unfolding over time—*scanpaths*—may support episodic remembering in a free recall task.

In the present study, we tackle this issue head on by using state-of-the-art scanpath similarity techniques, capable of quantifying the sequential encoding-recollection similarity (SERS) of scanpaths, and decomposing it into distinct spatio-temporal scanpath properties. The degree of SERS can then be examined over these scanpath properties during episodic retrieval, allowing us to examine and stratify the critical concept of scanpath replay.

**Figure 1.**
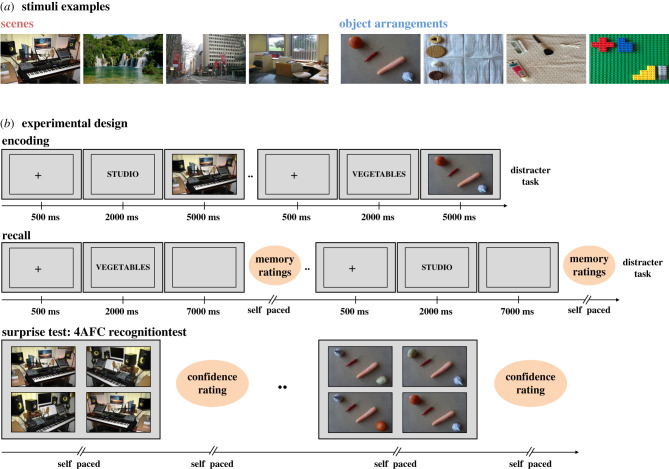
Encoding and recollection of scenes and object arrangements. (*a*) Example of stimuli images (scenes: studio, waterfalls, city street, office; object arrangements: vegetables, cookies, bathroom things, Lego). (*b*) Experimental design of the encoding phase, recall phase and surprise test. (Online version in colour.)

Participants encode images with associated verbal labels and subsequently recall each image while looking at a blank screen. In the recall phase, a particular image is cued by its verbal label, and the quality of the mnemonic content of each episodic reconstruction is subjectively rated with respect to vividness, spatial accuracy and overall recollection strength (figure 1*b*). To objectively assess the strength and quality of the mnemonic content during recall, our paradigm further combines those subjective ratings of memory quality with a subsequent surprise forced-choice recognition test (figure 1*b*). Finally, we use two types of image stimuli—scenes and object arrangements—to investigate if the SERS of scanpaths differentially contributes depending on the nature of the goal-relevant memory. The scenes comprise highly predictable spatial relations between meaningful scene elements (due to schematic and situational knowledge for the common scenarios), with relatively low demands on relational memory. The object arrangements comprise arbitrary spatial relations between their scene elements (objects), with relatively high demands on relational memory (figure 1*a*). These two types of stimuli allow us to examine the relative contribution of different scanpath reinstatements (such as fixation order and saccade direction) as a function of varying demands on relational memory [22].

## 2. Methods

### (a) Participants

Sixty-two healthy adults participated in the experiment. Two were removed owing to extensive data loss and technical problems, leaving sixty participants (34 females; mean age 25.2, s.d. 6.4). Power analyses [40] for bivariate measures of association indicated that our sample size should be sufficient to detect a large correlation (*r* = 0.5) and difference between means (*d* = 0.6) with 90% power (*α* = 0.05), which is in line with prior work on gaze reinstatement [17,27] and scanpath similarity [41]. All participants were fluent in Swedish and had normal or corrected-to-normal vision. Participants gave written informed consent and were compensated with a cinema voucher. All methods were conducted in accordance with the Swedish Act concerning the Ethical Review of Research involving Humans (2003:460) and the Code of Ethics of the World Medical Association (Declaration of Helsinki).

### (b) Data acquisition and materials

Gaze data were recorded from both eyes individually, using a SensoMotoric Instruments (SMI RED-M) eye tracker, running iView X 2.7 software and sampling at 120 Hz. A Dell Optiplex 755 PC presented stimuli using PsychoPy [42] on a 22-inch monitor with a resolution of 1680 × 1050 pixels. Participants were seated with their heads in a chin and forehead rest, 65 cm away from the monitor. Calibration and validation of gaze data was conducted prior to each participant's experimental session. Fixations were detected with the I2MC algorithm [43]. Stimuli comprised 18 scenes and 18 object arrangements that were presented in a frame that covered 80% of the monitor (figure 1*b*). See electronic supplementary material for data quality and general oculomotor data during encoding and recall. The scenes were taken from the MIT test set Cat2000 (http://saliency.mit.edu/datasets.html) [44] and the object arrangements were photographs shot for this particular study (available at https://osf.io/d9zng/).

The content of the scene images comprised an equal amount of indoor and outdoor global scenes, where the spatial relations among scene elements were highly congruent in respect to the image's semantic structure. The content of the object arrangement images comprised four separate objects from the same category, where the spatial relations between the objects were completely arbitrary in respect to the image's semantic structure. The 18 object arrangements all comprised unique spatial configurations (figure 1*a*; complete set of images available at https://osf.io/d9zng/). By contrast to the scene images, we thus had full experimental control over the spatial relations among individual scene elements (objects) in the object arrangement images.

### (c) Design and procedure

The experiment was divided into three phases: encoding, recall and surprise test (figure 1*b*). To conceal the true objective of the study, participants were told that the experiment concerned pupil dilation in relation to mental workload, and it was explained that their eyes were filmed for this matter.

### (d) Encoding phase

Participants encoded 36 images accompanied by a verbal label that preceded each image. The verbal label described the semantic content of the succeeding image (e.g. ‘studio’, ‘vegetables’). The images comprised two different types: scenes (*n* = 18) and object arrangements (*n* = 18) and were each presented for 5 s in randomized order. Participants were instructed to memorize each image as thoroughly as possible. When the encoding phase finished, participants engaged in a distracter task, where they were to count backward in steps of 3 from a randomly generated three-digit number for 12 s.

### (e) Recall phase

Participants recalled all 36 encoded images while looking at a blank screen. Participants were cued by the associated verbal label (preceding the blank screen) in a randomized order and were then instructed to recall and visualize the corresponding image in as much detail as possible while looking at the blank screen. The blank screen remained for 7 s (compared to encoding, this time was increased to compensate for the increased fixation durations that occur when looking at ‘nothing’ [13]). After each recollection, participants were to rate the quality of their recollection based on overall recollection strength, vividness and spatial accuracy. Overall recollection strength was rated on a scale 0–100% in relation to the statement ‘It was easy for me to remember the image’. Vividness was rated on a scale 0–100% in relation to the statement ‘My mental image was clear, vivid and detailed—almost as if I could see the image in front of me’ Spatial accuracy was rated on a scale of 0–100% in relation to the statement ‘I could indicate, with high spatial accuracy, where different objects/scene elements were located in the image’ (table 1). The three scores were highly correlated (*r* > 0.89, *p* < 0.001; see electronic supplementary material, figure S1) and based on a mean of those three ratings, a mnemonic content score was calculated for each image and participant.

**Table 1.** Mean values for the performance data during the recall phase and the surprise test, with standard deviations within brackets. The mnemonic content score represents the mean of the three subjective ratings (recollection strength, vividness, spatial accuracy). Only correct trials were considered for confidence, response time and gaze transitions between options (a measure of choice certainty [45]).

	total	scenes	object arrangements
performance data			
recall phase			
mnemonic content score (%)	58 (30)	66 (26)	49 (31)
surprise test			
accuracy (%)	85 (36)	95 (22)	75 (43)
response time (ms)	7699 (6746)	5583 (5672)	9815 (7065)
confidence (%)	78 (29)	90 (18)	66 (33)
gaze transitions between options	10.8 (7.4)	8.5 (5.2)	13.2 (8.4)

### (f) Surprise test

Participants completed four-alternative forced-choice (4AFC) recognition tests that covered all 36 images presented during encoding. Participants were not aware that they would engage in this test beforehand. The target image and three different distracter images were presented in the four quadrants of the screen, and participants were instructed to select the image they had encoded and recalled as accurately and quickly as possible. After each selection, participants rated how confident (0–100%) they were in their choice. For the scenes, the distracter images comprised: (1) a horizontal flip of the target image; (2) a similar lure image; and (3) a horizontal flip of the lure image. For the object arrangements, the distracter images comprised: (1) a lure image where two of the original objects had switched locations; (2) a lure image where one of the objects had been exchanged with another semantically congruent object; and (3) a lure image where one of the objects had been exchanged with the other semantically congruent object, and also the location of this new object had been switched with one of the other objects.

The present experimental design allowed us to collect *spontaneous eye movements* during recall, and to use the surprise test to evaluate the validity of the subjective ratings as a measure of recall success.

### (g) Statistical analyses

All data were analysed using generalized linear mixed-effects models (GLMMs; glmer of package lme4 [46]) and linear mixed-effects models (LMEMs), where participants and images were modelled as random effects (intercepts) and with random slopes for image types. In order to describe the model-fit of an independent variable, the deviance of the proposed model was contrasted against an unconditional null model, including only the intercept and the random factors. When building models with several independent variables, we used a backward selection approach, starting with a maximal model, which included all variables and their interactions. We then used likelihood-ratio tests to compare models and then step-by-step removed non-significant effects until no further model changes resulted in a significant likelihood-ratio test (*p* < 0.05). Models were fitted with restricted maximum-likelihood (REML) and Satterthwaite approximations were used to assess the significance of individual predictors.

## 3. Results

### (a) Behavioural results

We first sought to verify that the object arrangements were more demanding to recall than the scenes. As expected, mnemonic content was rated higher for scenes than object arrangements during recall, *β* = 0.165, (s.e. = 0.0317), *z* = 5.22, *p* < 0.001. For the surprise test, results revealed higher performance for scenes over four different measures of retrieval performance: response accuracy ($\chi_{1}^{2}=64.8$, *β* = 2.17, s.e. = 0.269, *z* = 8.05, *p* < 0.001), confidence ($\chi_{1}^{2}=51.0$, *β* = 0.175, s.e. = 0.0245, *z* = 7.14, *p* < 0.001), response time ($\chi_{1}^{2}=43.7$, *β* = 4132, s.e. = 625, *z* = 6.61, *p* < 0.001) and gaze transitions between options (a measure of choice certainty [45]; $\chi_{1}^{2}=53.6$, *β* = 4.62, s.e. = 0.631, *z* = 7.32, *p* < 0.001). Thus, these results demonstrate that the object arrangements, placing greater demand on relational memory, were indeed harder to recall than the scenes (table 1).

We next tested if the mnemonic content score during recall predicted subsequent retrieval performance during the surprise test. Results reveal that the mnemonic content score was a significant predictor of all four measures of retrieval performance: response accuracy ($\chi_{1}^{2}=6.17$, *β* = 0.645, s.e. = 0.260, *z* = 2.48, *p* = 0.013), confidence ($\chi_{1}^{2}=27.4$, *β* = 0.101, s.e. = 0.0192, *z* = 5.24, *p* < 0.001), response time ($\chi_{1}^{2}=6.53$, *β* = −1339, s.e. = 524, *z* = −2.55, *p* = 0.011) and choice certainty ($\chi_{1}^{2}=11.8$, *β* = −1.98, s.e. = 0.575, *z* = −3.44, *p* < 0.001; see electronic supplementary material, figure S2A–D). Higher subjective ratings during the recall phase thus predicted more accurate, confident, faster and certain responses during the subsequent surprise test. We therefore conclude that the mnemonic content score during recall is a representative index of the memory quality during episodic remembering.

### (b) Spatial reinstatement of static gaze patterns

In the next step, we examined the spatial encoding-recollection overlap of omnibus static position-based gaze, i.e. without considering the dynamic path that connects a sequence of eye movements unfolding over time. Here, we divided the screen into four areas of interest (AOIs), corresponding to the four quadrants of the screen, and then the proportional number of fixations within each of those four AOIs was compared over encoding and recall, for each image and participant. We found that the spatial locations of participants' gaze patterns broadly overlapped during encoding and recall, and this effect was pronounced for the more demanding object arrangements (electronic supplementary material, figure S3). Importantly, we also found that the degree of this spatial gaze reinstatement was predictive of our index of memory quality during recall (electronic supplementary material, figure S4). This corroborates what previous research has found [14,27,28], and provides further evidence that a spatial encoding-recollection overlap in viewing patterns supports episodic remembering. An exhaustive presentation of these analyses is available in the electronic *supplementary material* (where we also conduct the analyses for 8, 12 and 16 AOIs, to test the sensitivity of these analyses in respect to ‘spatial resolution’).

### (c) Sequential reinstatement of consecutive eye movements

To achieve the central goals of the present study, we next investigated SERS in scanpaths, and how the reinstatement of such ordered sequences of eye movements—scanpath replay—may support episodic remembering.

**Figure 2.**
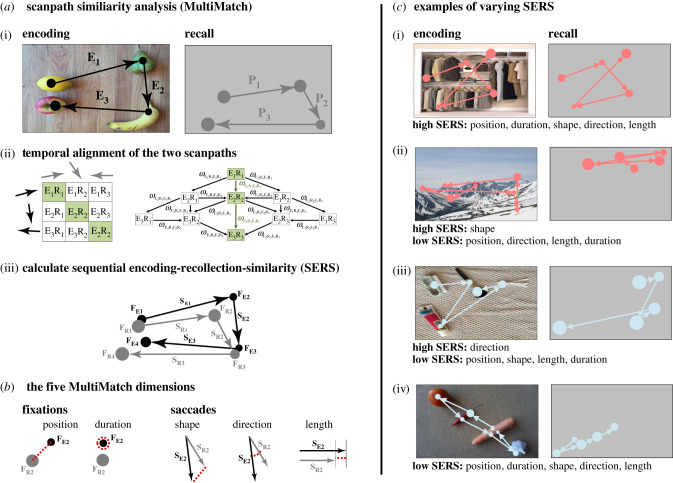
Illustrations of the method to capture sequential encoding-recollection similarity. (*a*) Overview of the MultiMatch scanpath similarity analysis. In the first panel (i), the scanpaths from encoding and recall to be compared are shown, where fixations are represented as dots and saccades as arrows between the fixations, and where larger dots represent longer fixation duration. In the second panel (ii), the basic principle behind the temporal alignment of the two scanpaths is illustrated. In the matrix to the left, each saccadic vector during encoding (E1–E3) is compared to each saccadic vector during recall (R1–R3) according to their shape. Using the Dijkstra algorithm [50], the optimal temporal alignment between the two scanpaths is then computed as the minimal cost—shortest path—from the upper left corner to the bottom right corner of the comparison matrix. All possible paths along with the cost for each transition (*ω*) between the matrix elements is outlined in the right figure. The minimal cost—shortest path—in this example (E_1_R_1_ to E_2_R_2_ to E_3_R_3_) is highlighted. Finally, the temporally aligned scanpaths from encoding and recall are shown superimposed in Euclidean space (iii) to illustrate how sequential encoding-recollection similarity (SERS) can be calculated for each individual fixation and saccade pairings over the five MultiMatch-dimensions. SERS for the complete scanpaths is then quantified as the average similarity over all the temporally aligned saccade (encoding: E_S1_–E_S4_; recall: R_S1_–R_S4_) or fixation (encoding: E_F1_–E_F4_; recall: R_F1_–R_F4_) pairings. (*b*) An illustration of the five MulitMatch dimensions of fixation position, fixation duration, saccade shape, saccade direction and saccade length for the temporally aligned fixation pairs F_E3_ − F_R3_ and saccade pairs S_E3_ − S_R3_. The numeric difference in SERS between each dimension is illustrated with a dotted line for each dimension separately. The fixation dimension of position relies on spatial coordinates in absolute space and quantifies how similar temporally aligned fixations are in respect to Euclidean distances, thus representing a similarity measure of fixation order. In contrast, the saccade dimensions of shape, direction and length rely on differences in relative space. The shape dimension quantifies how similar temporally aligned saccadic vectors are in overall geometric shape. The direction dimension quantifies how similar temporally aligned saccadic vectors are in geometric angle, thus representing a similarity measure of the particular heading of eye movements. The length dimension quantifies how similar temporally aligned saccades are in their absolute amplitude, irrespective of shape and direction. The fixation dimension duration does not rely on any spatial coordinates and quantifies how similar temporally aligned fixations are in their duration. (*c*) Examples of varying SERS: (i) complete SERS in respect to all five MM dimensions; (ii) relatively high SERS in shape, but during recall there is a dislocation in absolute space, large dissimilarities in saccadic angles, overall shorter saccades and overall longer fixation durations—therefore the SERS in all other MM dimensions are relatively low; (iii) high SERS in direction, but during recall there are dislocations in absolute space, disproportional saccadic lengths and overall longer fixation durations—therefore the SERS in all other MM dimensions are relatively low; (iv) low SERS over all five MM dimensions. (Online version in colour.)

To measure SERS, we used MultiMatch (MM), which was introduced as a method for comparing scanpaths over different spatio-temporal dimensions [47], and has subsequently been validated, applied, and evaluated against other scanpath comparison tools [36,47,48,49]. The basic principle is that the MM algorithm simplifies the two scanpaths under comparison (in this case, one from encoding and the other from recall) into virtualized ordered sequences of connected saccadic vectors (see [36,47] for a full explanation of the scanpath simplification procedure). The MM algorithm then temporally aligns the two scanpaths, so that particular saccade and fixation pairings from the two ordered scanpaths can be compared. The temporal alignment is achieved by matching the sequence of saccadic vectors from the two scanpaths according to their vector shape (length and direction) using a comparison matrix in which costs are drawn from differences in shape similarity between potential pairings. The MM algorithm then uses the Dijkstra [50] algorithm to find the shortest (minimal cost) path through the comparison matrix (from the top left corner to the bottom right—taking all possible routes into account). Now the two scanpaths can be sequentially aligned according to this path. Temporal alignment is thus a relative procedure which is not dependent on an equal number of saccades in the two scanpaths (or on equal encoding and recall times). The process of temporal alignment is indicated in figure 2*a* and is explained in full in [47]. SERS between the temporally aligned scanpaths can then be separately determined over five MM dimensions: (1) fixation position, (2) fixation duration, (3) saccade shape, (4) saccade direction and (5) saccade length (figure 2*b*). The five MM dimensions of SERS thus reflect different embodied connections between eye movements and memory, allowing us to not only investigate *if* a sequential reinstatement of consecutive eye movements supports episodic remembering, but also to scrutinize *how* qualitatively distinct aspects of such scanpaths influence this interplay—such as the sequential reinstatement of fixation order (MM dimension: fixation position) and pathway from one consecutive fixation to the next (MM dimension: saccade direction). As most of the MM dimensions do not rely on a reference frame in absolute space, the method is relatively unaffected by the documented spatial ‘offsets’ and ‘re-scaling’ of gaze patterns during recall [13,15–17,29], which are known to dilute the results when investigating scanpaths during a blank screen paradigm [36,47].

To determine if and how scanpaths from encoding become sequentially reinstated during recall, we first calculated SERS scores over all five MM dimensions (fixation: position, duration; saccades; shape, direction, length) by comparing the scanpaths produced by a participant during the encoding of an image, with those produced during recall of the same image. figure 2*c* for examples of varying SERS over the five MM dimensions. Second, by comparing the scanpaths produced by a participant during the encoding of an image with those produced during recall of all the other images from the same type (scenes, object arrangements), and then averaging across the scores calculated for all images, we acquired a baseline similarity score. This represents the average sequential similarity in scanpaths for each participant across images.

**Figure 3.**
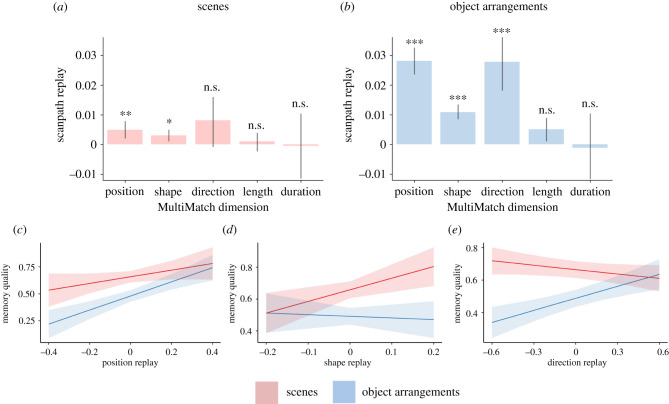
Scanpath replay over the five MM dimensions for the (*a*) scenes and (*b*) object arrangements. The measure of scanpath replay represents the difference between SERS and baseline similarity (a value greater than zero indicates scanpath replay). (*c*) The relationship between position replay and memory quality. (*d*) The relationship between shape replay and memory quality. (*e*) The relationship between direction replay and memory quality. Memory quality corresponds to the mnemonic content score during recall. Error bars and shaded areas denote 95% confidence intervals, *p < 0.05, **p < 0.01, ***p < 0.001. (Online version in colour.)

For the scenes, results revealed that the SERS score was significantly higher than the baseline similarity score for position ($\chi_{1}^{2}=6.83$, *β* = 0.0048, s.e. = 0.0018, *z* = 2.61, *p* = 0.009) and shape ($\chi_{1}^{2}=5.70$, *β* = 0.0030, s.e. = 0.0013, *z* = 2.39, *p* = 0.017). For the object arrangements, results revealed that the SERS score was significantly higher than the baseline similarity score for position ($\chi_{1}^{2}=148$, *β* = 0.0278, s.e. = 0.0023, *z* = 12.2, *p* < 0.001), shape ($\chi_{1}^{2}=55.8$, *β* = 0.0106, s.e. = 0.0014, *z* = 7.47, *p* < 0.001) and direction ($\chi_{1}^{2}=33.0$, *β* = 0.0269, s.e. = 0.0047, *z* = 5.74, *p* < 0.001). Length and duration were not significant predictors for either scenes or object arrangements ($\chi_{1}^{2}<2.30$, *p-*values > 0.13; figure 3*a,b*). These results thus show that position, shape and direction are scanpath properties that were sequentially reinstated during recall. However, significant SERS in direction was only observed for the object arrangements. The absence of SERS in length and duration demonstrates that amplitude of saccades (i.e. independent of direction and shape), and fixation durations are not sequentially retained during recall. However, as these properties typically depend on visual features from the image [51,52], which are absent during recall, there is no particular reason to expect SERS for either. Moreover, it is well known that fixation durations become atypically long when there are no external features to look at [13] and that saccades are frequently ‘contracted’ in size during recall [13,15,17,29]. See electronic supplementary material (electronic supplementary material, figure S5) for follow-up analyses that validate our methods of capturing sequential reinstatement and that scrutinize the results in relation to idiosyncratic behaviours.

### (d) Scanpath replay and episodic remembering

In the final step, we then tested whether the degree of scanpath replay for the significant MM dimensions (position, shape and direction) predicted our index of memory quality during the recall phase (the mnemonic content score). To quantify the degree of scanpath replay, we used a similar rationale as Wynn *et al.* [53], and subtracted the baseline similarity score from the SERS score for each MM dimension over each participant and image. The best model fit of memory quality revealed significant effects of image type ($\chi_{1}^{2}=26.48$, *β* = 0.169, s.e. = 0.033, *z* = 26.11, *p* < 0.001), position replay ($\chi_{1}^{2}=16.95$, *β* = 0.472, s.e. = 0.115, *z* = 4.12, *p* < 0.001), direction replay ($\chi_{1}^{2}=4.04$, *β* = 0.085, s.e. = 0.042, *z* = 2.01, *p* = 0.045) and significant interaction effects between image type and shape replay ($\chi_{1}^{2}=5.28$, *β* = 0.896, s.e. = 0.390, *z* = 2.30, *p* = 0.022), and between image type and direction replay ($\chi_{1}^{2}=16.05$, *β* = 0.337, s.e. = 0.084, *z* = 4.01, *p* < 0.001; see electronic supplementary material, table S2). Greater position replay predicted better memory quality independent of image type (figure 3*c*), whereas greater shape replay only predicted better memory quality for the scenes (figure 3*d*), and greater direction replay only predicted better memory quality for the object arrangements (figure 3*e*). See electronic supplementary material (electronic supplementary material, table S4, figure S6) for a follow-up analysis testing the specificity of this replay effect in relation to the temporal order of eye movements.

The results thus show that independent of the mnemonic content, looking at locations in a similar sequential order (i.e. position replay) during recall as during the original encoding predicts the quality of episodic remembering. This extends previous findings that encoding-recollection overlaps in fixation locations are relevant for episodic remembering [14,27,28] and indicates that the sequential order in which such fixations unfold also plays an active role in the recollection process. The interaction effects show that shape and direction replay contributed differently depending on the mnemonic content. Retaining the general spatial structure of consecutive saccades (i.e. shape replay) appears to be more important when reconstructing the more predictable spatial relations in the scene images. Conversely, retaining the particular heading of consecutive saccades (i.e. direction replay) appears to be more important when reconstructing the arbitrary spatial relations in the object arrangements.

### (e) Facilitating remembering or a consequence of the memory representation?

While the results indicate a functional role of scanpath replay in episodic reconstruction, an alternative explanation is that greater replay is a consequence of having a strong memory representation. To disambiguate between those two explanations, we sought to determine whether scanpath replay depends on how well the images are initially encoded into memory. Previous research has established that the cumulative number of fixations during encoding is a reliable index of how well a visual stimulus is encoded into memory [6–12]. Thus, to account for effects of encoding strength, we added the cumulative number of study fixations on each image into our model. The model was significantly improved ($\chi_{1}^{2}=24.90$, *p* < 0.001). All previous effects and interaction effects were significant also in this model, and as expected, the cumulative number of study fixations significantly predicted memory quality during recall (*β* = 0.008, s.e. = 0.002, *z* = 4.99, *p* < 0.001), which confirms the validity of this measure as an index of encoding strength. Interactions of number of study fixations and the other predictors (position replay, shape replay, direction replay, image type), were then stepwise added to the model. Importantly, none of those interactions significantly improved the model (*p*-values > 0.19). See electronic supplementary material, table S3*.* Thus, the reported effects of scanpath replay upon memory quality do not reduce to how well the images were originally encoded into memory. Instead, our results substantiate the idea that the replay of eye movements supports the reconstruction of inter-related and task-relevant mnemonic features into a spatio-temporal context.

## 4. Discussion

Corroborating previous research [14,27,28,31,32], we found that spatial reinstatement of gaze patterns predicts the fidelity of episodic remembering. State-of-the-art scanpath-similarity techniques [36] allowed us to move beyond omnibus static position-based gaze to examine sequential reinstatement of qualitatively different scanpath properties during episodic reconstructions. Of central importance, we provide evidence that the reinstatement of an ordered sequence of eye movements supports episodic remembering and that different spatio-temporal properties of the unfolding scanpaths differentially contribute depending on the nature of the goal-relevant memory. Finally, we provide evidence that these sequential reinstatement effects on episodic remembering are not simply a consequence of how well the event was originally encoded.

The claim that gaze behaviour supports episodic recollection dates back to (at least) Ulric Neisser and Donald Hebb. Neisser argued that eye movements are actively associated with memory reconstruction [24], and Hebb claimed that eye movements are necessary to assemble and organize ‘part images’ into a whole visualized image [23]. Such ideas were further developed in Noton and Stark's Scanpath Theory, which states that memories are stored in a ‘feature ring’, comprising visual features and the sequence of eye movements (scanpaths) linking them together [25,26]. Scanpath Theory holds that (a) eye movements during recall play out in the same sequential order as during encoding, and (b) such scanpath replay serves a functional role in episodic remembering. While a strong interpretation of Scanpath Theory, where memory is accompanied by an exact and full scanpath recapitulation, has been refuted in more recent research [17,22,39], there is extensive evidence that episodic remembering involves eye movements that broadly reproduce gaze patterns at encoding [13–17,27,29]. Thus, the first tenet of scanpath theory, that episodic remembering is accompanied by scanpath replay, is established in the literature, and gets further explicated in the present study. However, there is to date virtually no evidence for the second tenet, that scanpath replay serves a functional role when episodic information is recalled from memory in the absence of supporting visual input. Recent work has demonstrated that gaze reinstatements promote cortical reconstruction [29,30] and successful remembering [14,27,28,31–35]. However, these findings are all related to spatial reinstatement of static position-based gaze and shed no light upon the proposition that the sequential reinstatement of consecutive eye movements serve to reconstruct and bind spatio-temporal information into a coherent memory (but see ref. [54] for different temporal gaze dynamics during perception and mental imagery). Interesting recent research on recognition memory shows that a temporal replay of gaze patterns can support recognition performance [37,38,53]. Still, recognition tasks involve situations where all (or some) encoded information is available as a ‘copy cue’, and where visual information accumulates until sufficient evidence is available to solve the old/new-discrimination task. The purpose of visual exploration under such conditions is thus considerably different from a recall task, where the rememberer needs to mentally reconstruct the complete spatio-temporal properties of a prior event without supporting visual input (see ref. [6]). Here, we present direct evidence that a replay of sequentially ordered eye movements plays an active role in episodic reconstruction in a pure recall task.

Recently, Wynn and colleagues proposed that eye movements support active memory retrieval by broadly reinstating the spatio-temporal context based on current task demands and available cognitive resources [22]. The idea that the facilitatory role of gaze reinstatements increases with task demands has been supported by their studies on older adults [28] and task difficulty [53]. Further support comes from the present findings where more extensive spatial and sequential gaze reinstatement was seen during the reconstruction of the more demanding object arrangements as compared to the less demanding scenes, and that different spatio-temporal scanpath properties contributed differentially to successful recollection of those two types of images.

A vast amount of research has demonstrated that the hippocampus plays a critical role in binding information in space and time during memory formation, and it is assumed that the hippocampus stores an event index, pointing to the cortical representations of each aspect of the whole event (for a review see [55]). Remembering depends on a retrieval cue matching the hippocampal index, which in turn triggers pattern completion and the reactivation of the cortical traces (and thus the experience of remembering the original event). Based on this cortical-hippocampal interplay, a prominent view holds that the reconstruction of past events fundamentally relies on mental simulations that reinstate approximations of the sensorimotor processes that characterized the original event [56,57]. Successful episodic remembering is considered to depend on the overlap between available retrieval cues and stored memory traces [58]. Compatibility between the processes triggered by a retrieval cue and those engaged during encoding increases the likelihood of successful retrieval [58,59]. As previous research has demonstrated that gaze behaviour during encoding and retrieval are linked to activity in the hippocampus [3,60–65], and that gaze reinstatement correlates with neural reactivation [29,30], it is conceivable that the scanpath replay reported here is responsible for promoting cortical-hippocampal reactivation of visuospatial relations during episodic memory reconstruction. The successive reinstatement of consecutive eye movements may act as internally generated retrieval cues that continuously updates in an iterative fashion as the rememberer moves his/her eyes during the pattern completion process. In situations with high demands on relational memory, as for the recollection of the object arrangements, more specified retrieval cues would be required during the pattern completion process, as compared to the less demanding scenes, where the pattern completion process would be supported by more predictable scene semantics.

## 5. Conclusion

Spatial reinstatement of gaze patterns has proven important for episodic remembering. The present study extends this finding and provides direct evidence that the actual replay of an ordered sequence of eye movements unfolding over time facilitates episodic remembering, and that specific spatio-temporal scanpath properties differentially contribute depending on the nature of the goal-relevant memory.

## Data Availability

Data and materials are available on the OSF: https://osf.io/d9zng/. The data are provided in electronic supplementary material [66].
